# ELF3 Overexpression Contributes to the Malignant Transformation of HPV16 E6/E7-Immortalized Keratinocytes by Promoting CCNE2 Expression

**DOI:** 10.4014/jmb.2408.08041

**Published:** 2024-10-30

**Authors:** Yingping Zhu, Wenjuan Yang, Yulong Zhuang, Feifei Wang, Yanlu Ge, Jun Jiang, Danping Feng

**Affiliations:** 1 Department of Obstetrics and Gynecology, The First Affiliated Hospital of Zhejiang Chinese Medical University (Zhejiang Provincial Hospital of Chinese Medicine), P.R. China; 2 First School of Clinical Medicine, Zhejiang Chinese Medical University, P.R. China; 3 Department of Pediatrics, The First Affiliated Hospital of Zhejiang Chinese Medical University (Zhejiang Provincial Hospital of Chinese Medicine), 54 Youdian Road, Shangcheng District, Hangzhou, Zhejiang Province 310006, P.R. China

**Keywords:** Human papillomavirus genotype 16 E6/E7, keratinocytes, malignant transformation, E74 like ETS transcription factor 3, cyclin E2

## Abstract

Current cancer burden caused by persistent infection with human papillomaviruse genotype 16 (HPV16) cannot be ignored. The related mechanisms of oncoproteins E6 and E7 from HPV16 on keratinocyte malignant transformation need to be further elucidated. GSE3292 dataset analysis revealed the upregulation of ETS transcription factor 3 (ELF3) and cyclin E2 (CCNE2). To verify whether there is an interaction between ELF3 and CCNE2, E74 like ELF3 and CCNE2 expression profiles as well as their putative binding sites were analyzed using bioinformatics. Retroviruses encoding HPV16 E6 and E7 genes were used to induce immortalization of human foreskin keratinocytes (HFKs) in vitro. Dual luciferase reporters assay was used to verify the binding of ELF3 and CCNE2. The effect of ELF3 on the immortalized cells was investigated using CCK-8 assay, cell cycle analysis and western blot. ELF3 and CCNE2 presented overexpression patterns in head and neck squamous cell carcinoma. HPV16 E6/E7-expressing HFKs showed enhanced viability, accelerated cell cycle as well as upregulated ELF3 and CCNE2. ELF3 overexpression enhanced the activity of CCNE2 promoter. ELF3 silencing reduced viability, induced cell cycle arrest and suppressed expressions of CCNE2, E6 and E7 in HPV16 E6/E7-expressing HFKs. Downregulation of ELF3 played an inhibiting role in the malignant transformation of HPV16 E6/E7-immortalized HFKs by decreasing CCNE2 expression.

## Introduction

Human papillomaviruses (HPVs) are small non-enveloped DNA viruses that can induce hyperplastic epithelial lesions, often causing benign flat or protruding warts and occasionally, cancer [1]. It has been shown that about 5%of all human cancers, especially cervical and oropharyngeal cancers, are caused by persistent infection with high-risk HPV genotypes. The high-risk HPV genotype has HPV16, 18, 31, 33, 35, 39, *et al*. [2, 3]. Among these genotypes, HPV genotype 16 (HPV16) plays a major causative role in HPV-associated cancers, which is responsible for most HPV associated cancers [4]. Currently, significant progress has been made in HPV vaccination for the prevention and treatment of cervical cancer, however, the mechanisms by which HPVs drive the initiation of cancers still remain unclear and are needed to be further studied in hopes of providing new therapeutic interventions [5].

Ample evidence has shown the carcinogenicity of high-risk HPV genotypes results primarily from the activity of viral E6 and E7 [6]. These two oncoproteins are located in the early gene-coding region of HPVs and can be activated when the viral genome is integrated into the host genome to help viral replication and promote the HPV life cycle [7, 8]. During this process, E6 and E7 has been demonstrated to drive cells toward malignancy through multiple molecular pathways and also has been suggested to be required for the maintenance of cancer cell hallmarks, such as uncontrolled proliferation and angiogenesis, in HPV-mediated malignancies [9]. Keratinocytes function as the first line of defense in the epidermis against a variety of viruses including HPVs [10]. Meanwhile, E6 and E7 have been widely used to generate immortalized keratinocytes. Studies have found that PTPN14 degradation by high-risk human papillomavirus E7 limited keratinocyte differentiation and contributed to HPV-mediated oncogenesis [11]. HPV16-immortalized keratinocytes have been widely used to study possible mechanisms of viral oncoproteins on premalignant phenotypes and malignant transformation [12, 13].

E74 like ETS transcription factor 3 (ELF3) belongs to the epithelial-specific ETS transcription factor family that are critical for normal development as well as epithelial tissue differentiation and homeostasis [14]. ELF3 is broadly expressed in epithelial tissues and involved in epithelial differentiation through the regulation of gene transcription [15]. Aberrant expression of ELF3 has been found in various tumors and it acts as a promotor or suppressor for the malignant development of cancer cells [16]. It has been found that ELF3 was one of the genes up-regulated in malignant tumor cells and was able to up-regulate the expression of tumor genes associated with proliferation and anti-apoptosis [17]. Compared with HPV16-negative head and neck squamous cell carcinoma (HNSCC), the upregulation of ELF3 expression is observed in HPV16-positive HNSCC from the Gene Expression Omnibus (GEO). In addition, overexpression of ELF3 has been recently underlined to be required during the reprogramming of HPV16-infected keratinocyte subpopulations identified by single cell RNA sequencing [18]. At present, the effect of ELF3 on the evolution of HPV16-positive cancers is rarely reported.

Therefore, this study used models of HPV16-immortalized keratinocytes to explore the effect and underlying mechanism of ELF3 on malignant transformation of HFKs.

## Methods

### Bioinformatics Analysis

GSE3292 dataset, the gene expression profile associated with HPV in HNSCC, was retrieved from the GEO database (https://www.ncbi.nlm.nih.gov/geo/) for differential expression analysis. The differentially expressed genes (DEGs) were determined using the limma package, ggplot2 package in R software (version 4.0.2). Statistical significance was set at *p* < 0.05. Putative binding sites between ELF3 and cyclin E2 (CCNE2) were analyzed using the JASPAR database (http://jaspar.genereg.net/).

### Cell Culture

Neonatal primary human foreskin keratinocytes (HFKs) were obtained from Lonza (Catalog#: 00192907, Switzerland) and cultured in KGM-2 Keratinocyte Growth Medium-2 (CC-3107, Lonza, Switzerland) at 37°C with 5% CO_2_.

### Retroviruses and Plasmids

Recombinant pLXSN retroviral vectors either empty or encoding HPV16 E6 and E7 genes (pLXSN-16E6E7) were provided by Addgene (52394, USA). Full-length sequences of ELF3 were cloned in pRP[Exp]-mCherry-CMV vectors to construct ELF3-overexpressing plasmids which were obtained from VectorBuilder (China). Short hairpin RNA (shRNA) targeting ELF3 (shELF3; sense: 5’-GGCTAGAGGCTGAGACTTA-3’, antisense: 5’-TAAGTCTCAGCCTCTAGCC-3’) and shRNA negative control were synthesized by GenePharma (China). The promoter of CCNE2 was inserted into pGL4.10 vectors (Promega, USA) to construct the wild-type (wt) reporter plasmids and consensus sequence mutant (mut) reporter plasmids.

### Cell transduction and Dual Luciferase Reporters Assay

At passage two, HFKs were infected with pLXSN vectors or pLXSN-16E6E7 at 37°C for 24 h, as described previously [19]. Selection of infected cells was performed for 5 days following transduction using the culture medium containing with 100 μg/mL G418 (10131027, Thermo Fisher Scientific, USA). Surviving clones were pooled and amplified. Transfection of HFKs with ELF3-overexpressing plasmids or/and shELF3 carried out using Lipofectamine 3000 Transfection Reagent (L3000001, Thermo Fisher Scientific) for 48 h following the manufacturers’ protocol.

HFKs were co-transfected with ELF3-overexpressing plasmids/empty vectors and CCNE2-wt/ CCNE2-mut by Lipofectamine 3000 for 48 h. After that, Firefly and renilla luciferase activities within cells were determined using Dual-Luciferase Reporter Assay System (E1910, Promega) according to the manufacturer’s instructions. Data were measured using a Veritas Microplate Luminometer (Turner Biosystems, USA).

### Cell Viability Evaluation

After cell transduction, HFKs were transferred and cultured in 96-well plates (3 × 10^3^ cells/well) for 24 h at 37°C in a 5% CO_2_ incubator. Next, 10 μL cell counting kit-8 (CCK-8) solution (40203ES60, Yeasen, China) was added into each well, followed by a 4-h incubation. The absorbance (450 nm) was determined using a microplate spectrophotometer (Multiskan FC, Thermo Fisher). Cell viability was calculated as follows: Cell viability (%) = (A_experimental_-A_blank_)/(A_control_-A_blank_) × 100%.

### Cell Cycle Assay

The effect of ELF3/CCNE2 axis on cell cycle of HFKs was detected using Cell Cycle and Apoptosis Analysis Kit (C1052, Beyotime, China). Briefly, 2 × 10^5^ cells were washed with pre-cooled phosphate-buffered saline (ST476, Beyotime) and then, were fixed with 70% ethanol (MFCD00003568, Sigma-Aldrich, USA) at 4°C for 2 h. After removal of the supernatant, the cells in each tube were incubated with 0.5 mL propidium iodide solution containing RNase A at 37°C for 30 min without light. Cell cycle analysis was performed by CytoFLEX flow cytometer (Beckman Coulter, USA) with CytExpert 2.3 software.

### Western Blot

Cell homogenate was prepared with RIPA Lysis Buffer (89901, Thermo Fisher Scientific,) containing protease and phosphatase inhibitors (78445, Thermo Fisher Scientific). Protein concentration in the supernatant was quantified with BCA Protein Assay Kit (PC0020, Solarbio, China). Equal amounts of protein (20 μg) were separated using 10% SDS-PAGE and transferred to nitrocellulose membranes (IB23001, Thermo Fisher Scientific). After blocking with 5% non-fat milk (GC310001, Servicebio, China) at room temperature (RT) for 1 h, the separated blots were incubated with primary antibodies against E6 (sc-460, 17 kDa, Santa Cruz Biotechnology, USA), E7 (ab308180, 11 kDa, Abcam, UK), CCNE2 (ab32103, 45 kDa, Abcam) and loading control GAPDH (ab8245, 37 kDa, Abcam) at 4°C overnight, followed by a 2-h hybridization with horseradish peroxidase-conjugated secondary antibodies (abs20039, abs20147, Absin, China) at RT. The immunoblots were visualized using ECL luminescence reagent (abs920, Absin), and protein signals were densitometrically analyzed by Image Quant LAS 4000 system (GE Healthcare, USA).

### RNA Isolation and Quantitative Real-Time Reverse Transcription Polymerase Chain Reaction (qRT-PCR)

Total RNA from HFKs was isolated with TRIzol reagent (15596026, Thermo Fisher Scientific), and RNA concentration was determined by spectrophotometer (Cary5000, Agilent, USA). Then, 10 μg of total RNA was reverse-transcribed into complementary DNA (cDNA) with the application of SuperScript III ReverseTranscriptase Kit (18080093, Thermo Fisher Scientific). qRT-PCR was conducted in a thermal cycler (iCycler, Bio-Rad, USA) with Universal SYBR Green Supermix (1725120, Bio-Rad). Relative mRNA expressions were determined using 2^-ΔΔCt^ method and GAPDH was used as an endogenous control. Primer sequences used for this reaction are as follows (5’-3’): E6 (forward: CAGGAGCGACCCAGAAAGTT, reverse: GCAGTAACTGTTGCTTGCAGT), E7 (forward: CCGGACAGAGCCCATTACAA, reverse: TTTGTACGCACAACCGAAGC), ELF3 (forward: GGC CGATGACTTGGTACTGAC, reverse: GCTTGCGTCGTACTTGTTCTTC), CCNE2 (forward: TCAAGACGA AGTAGCCGTTTAC, reverse: TGACATCCTGGGTAGTTTTCCTC) and GAPDH (forward: TTTTGCGTC GCCAGCC, reverse: ATGGAATTTGCCATGGGTGGA).

### Statistical Analysis

Data are expressed as mean ± standard error of the mean from at least three independent experiments. Graphpad Prism 8.0 (GraphPad Software Inc., USA) was employed for all statistical analysis. Comparisons between two groups or among multiple groups were analyzed using independent samples *t*-test or one-way analysis of variance. Results at a significance level of *p* < 0.05 were considered statistically significant.

## Results

### ELF3 and CCNE2 Presented Overexpression Patterns in HNSCC

**Fig. 1:**
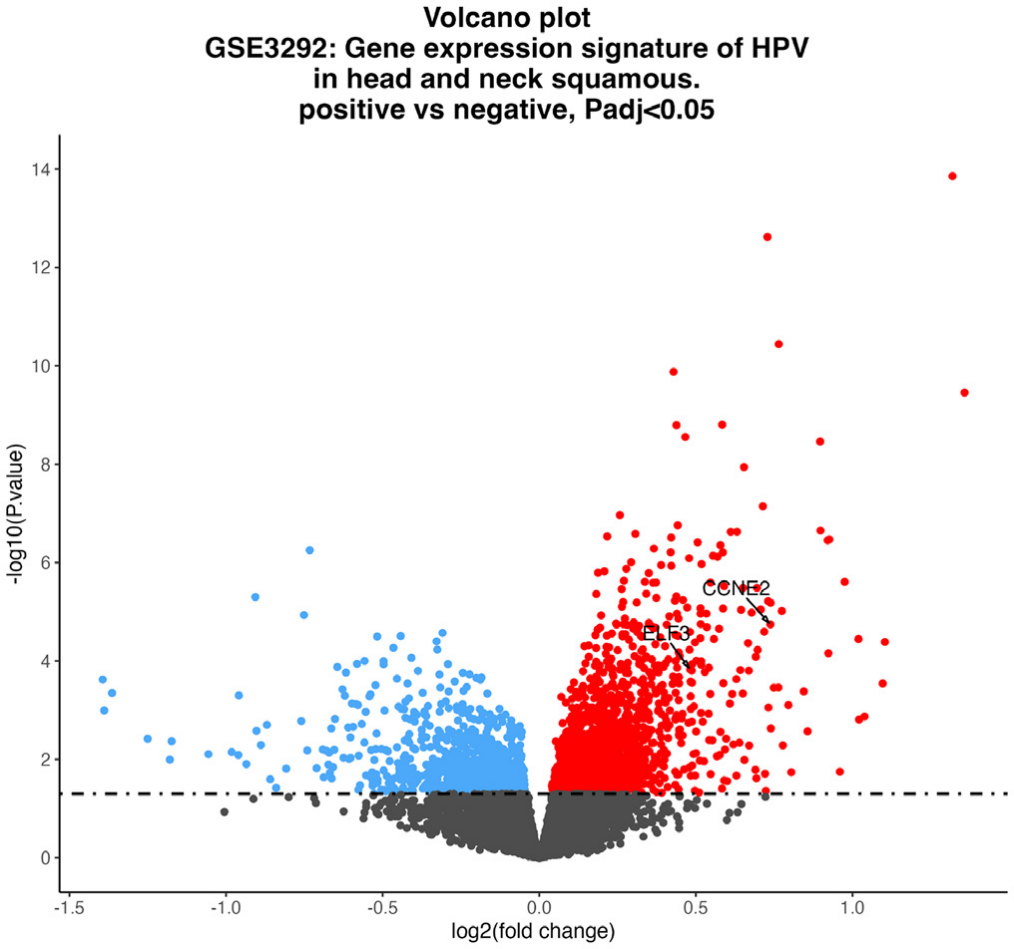
Expression profiles of ELF3 and CCNE2. Volcano plot of differential expression of genes associated with HPV in head and neck squamous cell carcinoma.

Based on the GSE3292 dataset, we analyzed differential expression of genes in HPV16-positive HNSCC. It was demonstrated by volcano plot that there were 1594 and 1074 up-regulated and downregulated genes in the samples, respectively, and that the expression of ELF3 and CCNE2 was upregulated (Fig. 1, *p* < 0.05).

### HPV16 E6/E7-Expressing HFKs Showed Increased Viability and Accelerated Cell Cycle

**Fig. 2:**
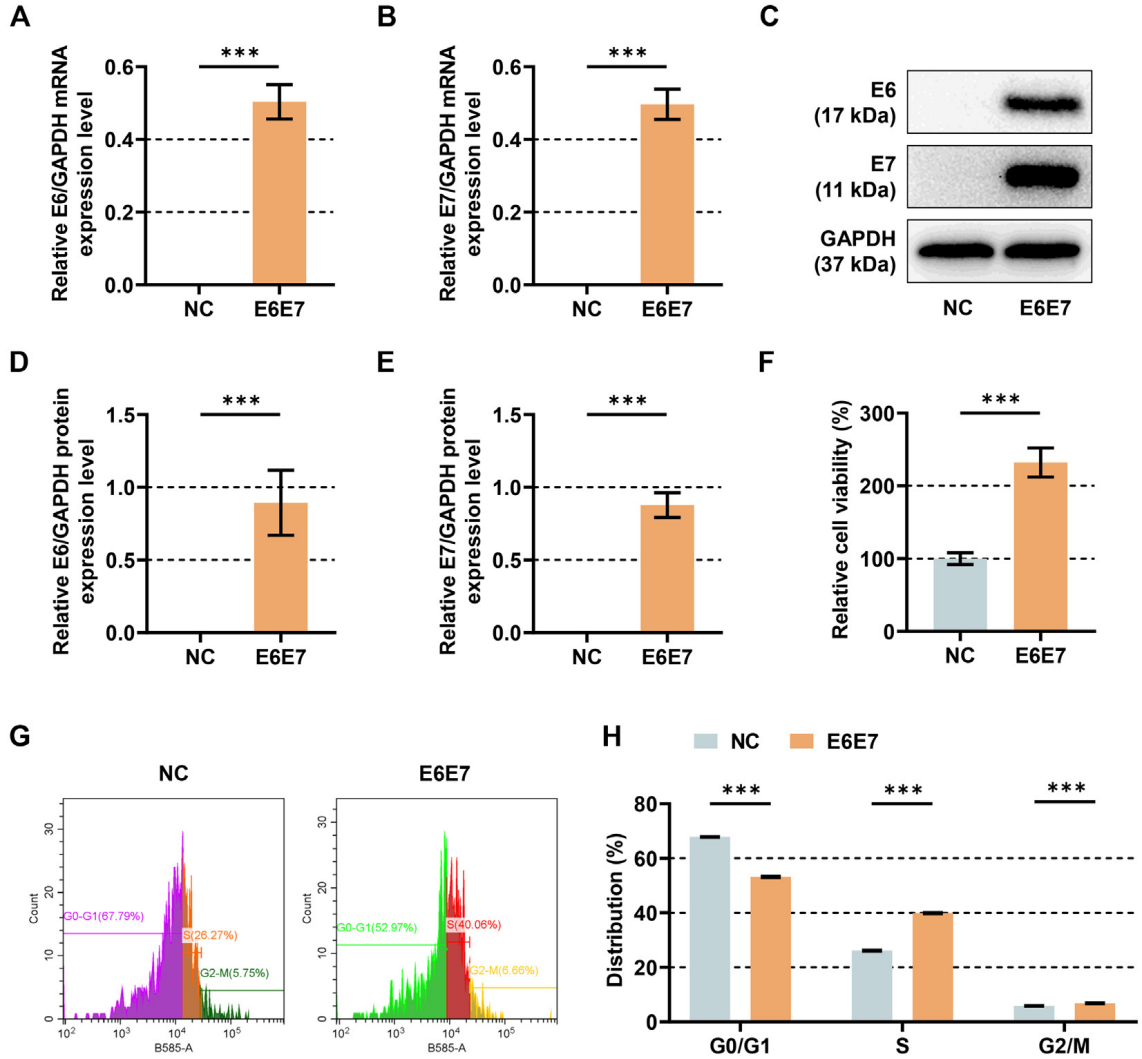
Effects of HPV16 E6/E7 overexpression on viability and cell cycle of HFKs. (A-B) Analysis of HPV16 E6/ E7 expressions in HFKs by qRT-PCR after transduction with retroviral control (pLXSN) or pLXSN-16E6E7. (C-E) Analysis of HPV16 E6/E7 expressions in HFKs by western blot after transduction with retroviral control or pLXSN-16E6E7. (F) CCK-8 assay was used to assess the viability of HFKs transduced with retroviral control or pLXSN-16E6E7. (G-H) Flow cytometry was performed to detect the cell cycle of HFKs transduced with retroviral control or pLXSN-16E6E7. GAPDH functioned as an endogenous control. Data are shown as mean ± standard deviation. ***P < 0.001. Abbreviation: HPV16, human papillomavirus genotype 16; HFKs, human foreskin keratinocytes; pLXSN-16E6E7, pecombinant pLXSN retroviral vectors encoding HPV16 E6 and E7 genes; qRT-PCR, quantitative real-time reverse transcription polymerase chain reaction; CCK-8, cell counting kit-8.

It is established that E6 and E7, as oncoproteins of HPV16 are associated with immortalization and malignant transformation of keratinocytes through regulation of cell cycle, cell proliferation and cell differentiation etc [20]. To study the effect of ELF3 and CCNE2 on HPV-mediated epithelial carcinogenesis, HFKs were subjected to infection with pLXSN-16E6E7 retrovirus or pLXSN retrovirus. After that, E6 and E7 expressions were determined by qRT-PCR and western blot. As shown in Fig. 2A-2E, both mRNA and protein expressions of E6 and E7 were increased in the HPV16 E6/E7-expressing cells compared to the negative control (*p* < 0.001). Subsequently, the results of CCK-8 assay showed that transduction with HPV16 E6/E7 enhanced the viability of HFKs (Fig. 2F, *p* < 0.001). In addition, there was a decrease in G0/G1 distribution and increases of S and G2/M distributions in cell cycle of HFKs after transduction with HPV16 E6/E7 (Fig. 2G and 2H, *p* < 0.001).

### ELF3 and CCNE2 Were Overexpressed in HPV16 E6/E7-Expressing HFKs

**Fig. 3:**
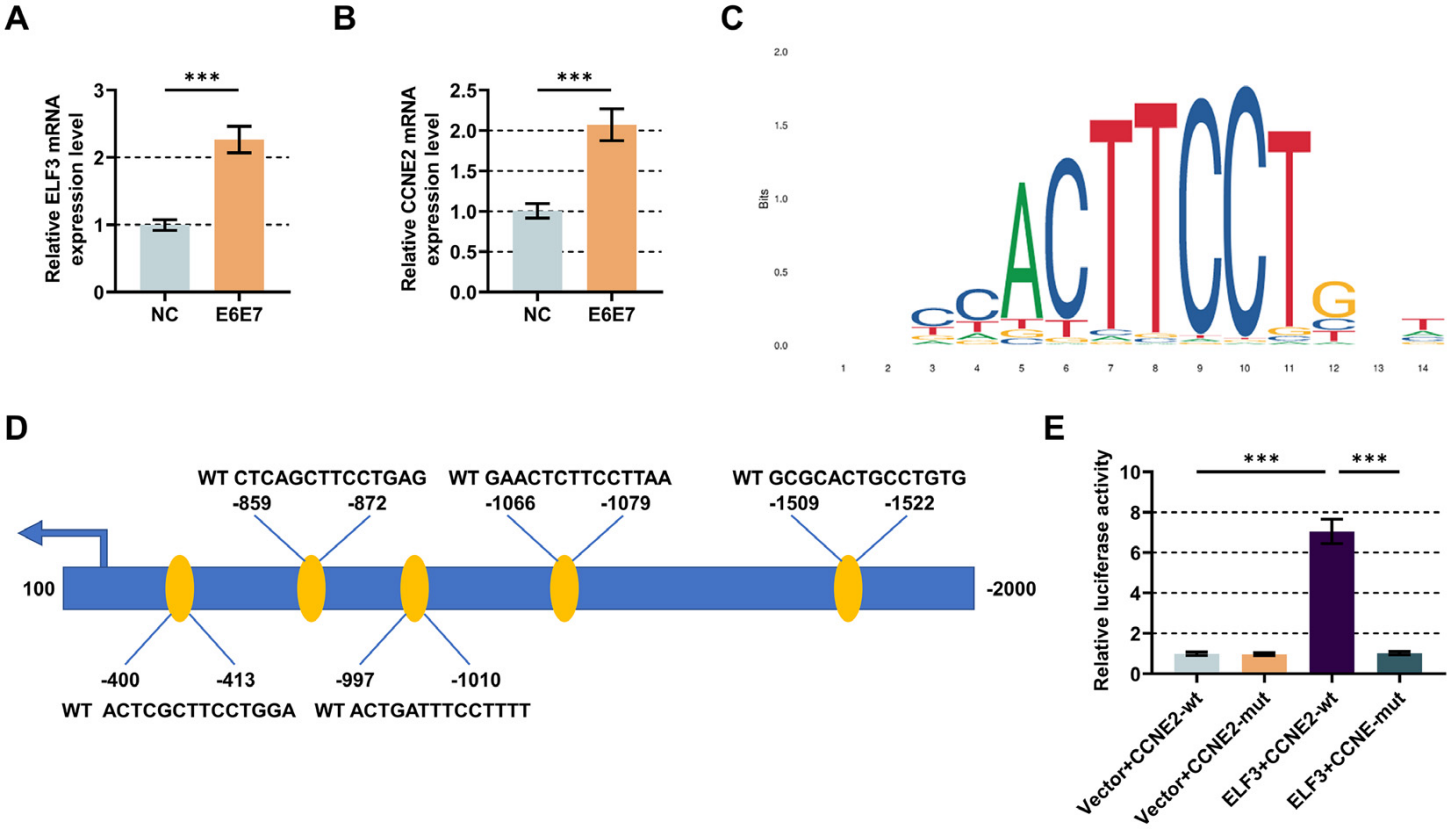
ELF3 and CCNE2 expressions in HFKs expressing HPV16 E6/E7 as well as their interaction. (**A-B**) Analysis of ELF3 and CCNE2 expressions in HFKs by qRT-PCR after transduction with retroviral control or pLXSN-16E6E7. GAPDH functioned as an endogenous control. (**C-D**) Bioinformatics analysis of putative binding sites between ELF3 and CCNE2. (**E**) Dual luciferase reporters assay was performed to verify the combination between ELF3 and CCNE2 in HFKs. Data are shown as mean ± standard deviation. ***P < 0.001. Abbreviation: ELF3, E74 like ETS transcription factor 3; CCNE2, cyclin E2; CCNE2-wt, wild-type reporter plasmids encoding the promoter of CCNE2; CCNE2-mut, consensus sequence mutant of CCNE2 promoter reporter plasmids.

In contrast to pLXSN retrovirus-infected HFKs, it was discerned that mRNA expressions of ELF3 and CCNE2 were increased in HFKs in the presence of HPV16 E6/E7 (Fig. 3A and 3B, *p* < 0.001).

### ELF3 Overexpression Enhanced the Activity of CCNE2 Promoter

Next, we investigated the interaction between ELF3 and CCNE2 promoter. Through bioinformatics analysis, ELF3 was predicted to contain binding sites for CCNE2, and found five sites in 400-413, 859-872, 997-1010, 1066-1079 and 1509-1522 (Fig. 3C and 3D). Dual-luciferase reporter assay was used to better define the targeted regulation between ELF3 and CCNE2. The results showed that cellular luciferase activity was significantly increased with both CCNE2-WT and ELF3, with no significant difference in both CCNE2-MUT and ELF3, which showed that ELF3 overexpression promoted relative luciferase activity in CCNE2-wt-transfected HFKs (Fig. 3E, *p* < 0.001). It is indicated that ELF3 positively regulates CCNE2 expression by activating the promoter of CCNE2.

### ELF3 Silencing Decreased Viability, Induced Cell Cycle Arrest and Suppressed Expressions of CCNE2, E6 and E7 in HPV16 E6/E7-Expressing HFKs

**Fig. 4:**
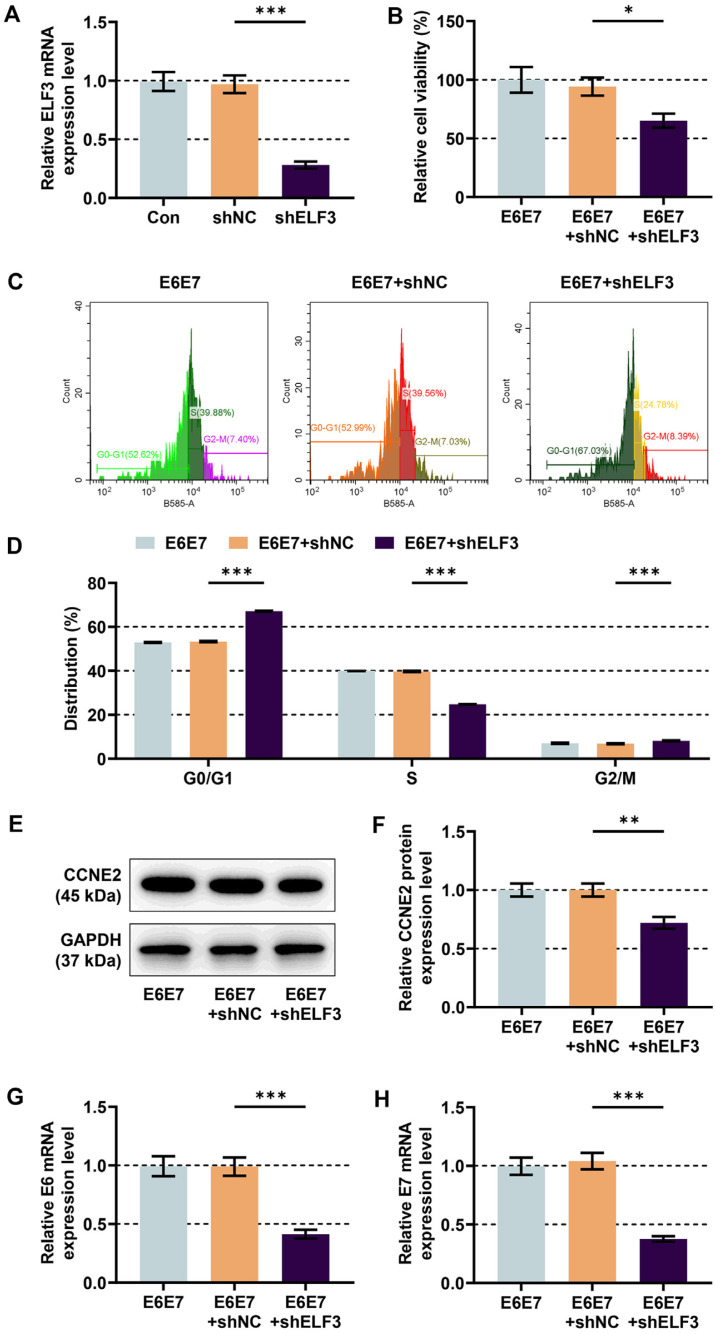
Effects of the ELF3/CCNE2 axis on malignant transformation of HFKs. (**A**) Analysis of ELF3 expression in HFKs by qRT-PCR after transfection with shELF3 or shNC. (**B**) CCK-8 assay was used to assess the viability of HPV16 E6/E7- expressing HFKs transfected with shELF3 or shNC. (**C-D**) Flow cytometry was performed to detect the cell cycle of HPV16 E6/ E7-expressing HFKs transfected with shELF3 or shNC. (**E-F**) Analysis of CCNE2 expression in HPV16 E6/E7-expressing HFKs by western blot after transfection with shELF3 or shNC. (**G-H**) Analysis of HPV16 E6/E7 expressions in HPV16 E6/E7- expressing HFKs by qRT-PCR after transfection with shELF3 or shNC. GAPDH functioned as an endogenous control. Data are shown as mean ± standard deviation. *P < 0.05, **P < 0.01, ***P < 0.001. Abbreviation: shELF3, short hairpin RNA (shRNA) targeting ELF3; shNC, shRNA negative control.

To further understand whether ELF3 plays a role in viability and cell cycle of HPV16 E6/E7-expressing HFKs through the regulation of CCNE2, HFKs were subjected to transfection with shELF3. Compared to the negative control, the mRNA expression of ELF3 in the cells was inhibited by shELF3 (Fig. 4A, *p* < 0.001). In the HPV16 E6/E7-expressing cells, it was observed a decrease in cell viability after ELF3 silencing (Fig. 4B, *p* < 0.05). Concomitantly, ELF3 silencing induced cell cycle arrest in the HPV16 E6/E7-expressing cells by expanding G0/G1 phase and shortening S and G2/M phases (Fig. 4C and 4D, *p* < 0.001). As demonstrated in Fig. 4E-4F, the increased protein expression of CCNE2 by HPV16 E6/E7 in HFKs was restrained following ELF3 silencing (*p* < 0.01). Compared to HPV16 E6/E7-expressing HFKs, moreover, following ELF3 silencing suppressed E6 and E7 mRNA expressions in the cells (Fig. 4G and 4H, *p* < 0.001).

## Discussion

It is well accepted that dysfunction of genes, such as miRNA and mRNA, is implicated in the initiation and progression of various cancers including HPV-associated cancers [21]. Owing to the advance of high-throughput RNA sequencing, increasing differentially expressed genes have been found to be associated with HPV16 infections, which provides new perspectives for the regulation mechanisms of HPV16 in keratinocytes at the preneoplastic stage [22]. To our best knowledge, this is the first study that reveals a triggering role of ELF3 in oncogenesis by regulating cell cycle-related CCNE2 on the basis of HPV16 E6/E7-expressing HFKs.

In keratinocytes from different anatomic parts, productive HPV16 infection can induce cell hyperproliferation, manifested by uncontrolled cell division and increased DNA replication, and these scenarios have been suggested to potentially cause epidermis-originated malignancies [2]. As a well-known oncoprotein, HPV16 E6 has been previously reported to induce tumor suppressor p53 degradation and thereby inhibit DNA damage and apoptosis [23]. However, recent studies have highlighted that E6, mainly in alternatively spliced forms, contributes to immortalization independently of p53 in keratinocytes [24, 25]. Besides, E7 from high-risk HPVs acts as a restriction protein to bind to retinoblastoma (pRB) and disassociate pRB from E2F, resulting in the re-entry of cell cycle [20]. In this study, we successfully transduced HPV16 E6/E7 into HFKs by pLXSN-based retroviruses to induce cell immortalization. Of note, increased S and G2/M phases as well as reduced G0/G1 phase were determined in the immortalized cells. It is known that the lengthening of the S phase plays a maintaining role in cell cycle, which leads to continuous amplification in the epithelial differentiation of the supra-basal keratinocytes in the context of high-risk HPV infection due to the dependence of HPV life cycle on replication enzymes in host cells [26].

**Fig. 5:**
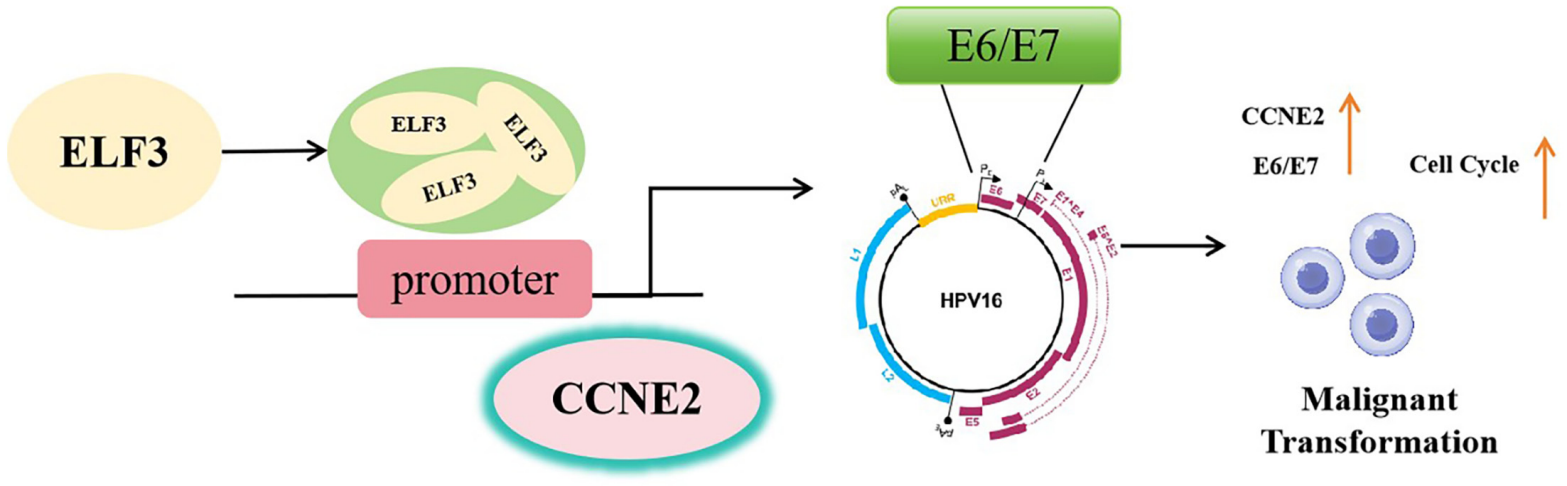
ELF3 regulated the malignant transformation of HPV16 E6/E7-immortalized keratinocytes through the regulation of CCNE2.

At present, vaccines for the prophylaxis of some HPV genotypes are available, but they are ineffective in HPV-infected individuals [27]. Therefore, given the importance of E6 and E7 continuous expressions in driving several human malignancies like cervical cancer, alternative strategies that block oncogenic activities of E6 and E7 have promise in suppressing the infected keratinocytes towards neoplastic progression. In recent years, ELF3 has been frequently thought as a regulator for cell cycle and epithelial differentiation to participate in the development of several cancers such as oral squamous cell carcinoma, non-small cell lung cancer and prostate cancer [28-30]. In the study of HPV 16 positive cervical intraepithelial neoplasia, downregulation of ELF3 has been found to inhibit the squamous differentiation of human cervical dysplasia cells in vitro [31]. In this study, we determined that ELF3 and CCNE2 were overexpressed in HPV16 E6/E7-expressing HFKs. But the relationship between ELF3 and CCNE2 has not been investigated. As a transcription factor, ELF3 was confirmed to bind to CCNE2 and promote the activity of CCNE2 promoter in the present study. As we known, DNA replication in cell cycle is precisely orchestrated to maintain genome stability. In normal cell cycles, E2F-mediated cyclin E proteins associate with cyclin-dependent kinase 2 (CDK2) to promote G1/S transition and thereby regulate cell cycle progression and DNA replication [32]. It has been shown that the amplification of CCNE2 can induce oncogenic activation of cyclin E/CDK2 complex, which consequently leads to genomic instability and malignancy [33]. Upregulation of CCNE2 expression is found in a variety of cancers and correlated with tumor progression [34, 35]. Additionally, inhibiting the expression of CCNE2 has been demonstrated to facilitate cell cycle arrest at the G1 phase and thereby decrease colorectal cancer cells in vitro and in vivo [36]. A recent evidence has showed an increase of CCNE2 expression in HPV16 E7 transgenic mice undergone chronic estrogen treatment, and this condition is accompanied by the development of cancerous cervical tissues [37]. Based above-mentioned findings, it is suggested that CCNE2 may play an important role in the malignant development of HPV16-associated cancers by regulating cell cycle. Here, we subsequently determined that ELF3 regulated the malignant transformation of HPV16 E6/E7-immortalized keratinocytes through the regulation of CCNE2 (Fig. 5). And ELF3 silencing resulted in cell cycle arrest in HPV16 E6/E7-expressing HFKs with a concomitant decline in CCNE2, E6, and E7 expressions. Therefore, it is indicated that downregulation of ELF3 reverses the HPV16 E6/E7-induced immortalization of HFKs through the inhibition of CCNE2. Nevertheless, this regulatory mechanism needed to be further validated in animal models in the future.

## Conclusion

In conclusion, our current findings provide new evidence that ELF3 overexpression facilitates the malignant transformation of HPV16 E6/E7-immortalized keratinocytes by promoting CCNE2 expression. In terms of this, we support that targeting ELF3 is a potential therapeutic strategy to inhibit oncogenic activities of E6 and E7 in HPV16-infected patients, thereby reducing the risk of carcinogenesis.

## Supplemental Materials

Supplementary data for this paper are available on-line only at http://jmb.or.kr.
